# Advancing Predictive Models for Early Detection of Alzheimer's Disease: Integrating Speech Characteristics and Cardiovascular Risk Factors for Enhanced ATN Biomarker Status Prediction

**DOI:** 10.1002/alz70856_103534

**Published:** 2025-12-25

**Authors:** Dishaa Sinha, Kushagra Soni, Ishan Durve, Vaibhav Soni, Hussein Mehmet, Nishat Tahira, Harshita Sinha, Junaid Chaudhary, Iman Kashif, Ivan Koychev

**Affiliations:** ^1^ University of Oxford, Oxford, Oxfordshire, United Kingdom; ^2^ Innoflexion, Bangalore, Karnataka, United Kingdom; ^3^ University of Sheffield, Sheffield, South Yorkshire, United Kingdom; ^4^ Nottinghamshire Healthcare NHS Foundation Trust, Nottingham, Nottinghamshire, United Kingdom; ^5^ West London NHS Trust, London, London, United Kingdom; ^6^ Fergusson College, Pune, Maharashtra, India; ^7^ Addenbrookes Hospital, Cambridge University Hospitals NHS Foundation Trust, Cambridge, Cambridgeshire, United Kingdom; ^8^ Imperial College London, London, London, United Kingdom

## Abstract

**Background:**

Alzheimer's disease (AD) is a neurodegenerative disorder characterized by progressive cognitive decline and memory impairment. It presents a significant healthcare challenge, particularly in aging populations. Early diagnosis is essential for timely intervention, which can slow disease progression and improve outcomes. Traditional diagnostic methods, including neuroimaging and invasive biomarker analyses, are costly and often inaccessible for routine monitoring. As a result, non‐invasive, affordable biomarkers are gaining attention for the early detection of AD. The ATN framework provides a biological definition of AD based on Amyloid (A), Tau (T), and Neurodegeneration (N) biomarkers, independent of clinical symptoms. Predicting ATN pathologies using speech characteristics and cardiovascular factors presents a novel, scalable approach to early diagnosis and management.

**Method:**

Data from 1,011 participants was aggregated from multiple clinical datasets through Bio‐Hermes, encompassing medical history and test result scores. Biomarker thresholds for Aβ42, Aβ40, pTau‐217, and NfL were used to classify participants into five ATN groups. Boolean flags for A, T, and N enabled classification into cognitively healthy, MCI, AD, and non‐AD pathology categories. Four XGBoost models were iteratively developed:

1. **Baseline Model**: Utilized demographic and lifestyle features (age, gender, education, comorbidities, genetics, and family history) with hyperparameter tuning and feature selection.

2. **Cardiovascular Model**: Variables like heart rate, cholesterol, and diabetes (Baseline +Cardiovascular).

3. **Speech Model**: Features like word recall, object recall, and speaking rate (Baseline + Speech).

4. **Combined Model**: Integrated baseline, cardiovascular, and speech features, excluding low‐performing features.

**Result:**

K‐fold cross‐validation was used for reliable accuracy scores. The baseline model achieved an ROC‐AUC of 0.9103 and accuracy of 0.7097. Cardiovascular model achieved an ROC‐AUC to 0.9254 and accuracy to 0.7608. Speech model achieved an ROC‐AUC of 0.9416 and accuracy of 0.7869. The combined model achieved the best results, with an ROC‐AUC of 0.9539 and accuracy of 0.8260.

**Conclusion:**

Incorporating cardiovascular and speech features significantly improved model performance for predicting ATN pathology. These findings underscore the potential for non‐invasive, scalable tools to detect early AD‐related changes, reducing dependence on invasive diagnostic tests.

